# Comparative Genomic, MicroRNA, and Tissue Analyses Reveal Subtle Differences between Non-Diabetic and Diabetic Foot Skin

**DOI:** 10.1371/journal.pone.0137133

**Published:** 2015-08-28

**Authors:** Horacio A. Ramirez, Liang Liang, Irena Pastar, Ashley M. Rosa, Olivera Stojadinovic, Thomas G. Zwick, Robert S. Kirsner, Anna G. Maione, Jonathan A. Garlick, Marjana Tomic-Canic

**Affiliations:** 1 Human Genetics and Genomics Graduate Program in Biomedical Sciences, University of Miami Miller School of Medicine, Miami, FL, United States of America; 2 Wound Healing and Regenerative Medicine Research Program, Department of Dermatology and Cutaneous Surgery, University of Miami Miller School Of Medicine, Miami, FL, United States of America; 3 University of Miami Hospital, UM Health System, Miami, FL, United States of America; 4 Cell, Molecular, Developmental Biology, Tufts University, Sackler School of Graduate Biomedical Sciences, Boston, MA, United States of America; 5 Department of Oral and Maxillofacial Pathology, Oral Medicine and Craniofacial Pain School of Dental Medicine, Tufts University, Boston, MA, United States of America; University Hospital Hamburg-Eppendorf, GERMANY

## Abstract

Diabetes Mellitus (DM) is a chronic, severe disease rapidly increasing in incidence and prevalence and is associated with numerous complications. Patients with DM are at high risk of developing diabetic foot ulcers (DFU) that often lead to lower limb amputations, long term disability, and a shortened lifespan. Despite this, the effects of DM on human foot skin biology are largely unknown. Thus, the focus of this study was to determine whether DM changes foot skin biology predisposing it for healing impairment and development of DFU. Foot skin samples were collected from 20 patients receiving corrective foot surgery and, using a combination of multiple molecular and cellular approaches, we performed comparative analyses of non-ulcerated non-neuropathic diabetic foot skin (DFS) and healthy non-diabetic foot skin (NFS). MicroRNA (miR) profiling of laser captured epidermis and primary dermal fibroblasts from both DFS and NFS samples identified 5 miRs de-regulated in the epidermis of DFS though none reached statistical significance. MiR-31-5p and miR-31-3p were most profoundly induced. Although none were significantly regulated in diabetic fibroblasts, miR-29c-3p showed a trend of up-regulation, which was confirmed by qPCR in a prospective set of 20 skin samples. Gene expression profiling of full thickness biopsies identified 36 de-regulated genes in DFS (>2 fold-change, unadjusted p-value ≤ 0.05). Of this group, three out of seven tested genes were confirmed by qPCR: SERPINB3 was up-regulated whereas OR2A4 and LGR5 were down-regulated in DFS. However no morphological differences in histology, collagen deposition, and number of blood vessels or lymphocytes were found. No difference in proliferative capacity was observed by quantification of Ki67 positive cells in epidermis. These findings suggest DM causes only subtle changes to foot skin. Since morphology, mRNA and miR levels were not affected in a major way, additional factors, such as neuropathy, vascular complications, or duration of DM, may further compromise tissue’s healing ability leading to development of DFUs.

## Introduction

In 2010 the prevalence of diabetes mellitus (DM) was estimated around 6.4% of the adult world population [1] and its prevalence is predicted to increase to 7.7% by 2030. This suggests that over the next 15 years an additional 150 million adults will develop DM [1]. DM has numerous associated complications and approximately a third of patients with DM have cutaneous manifestations [2]. These include chronic foot ulcers, dermopathy, necrobiosis lipoidica, and calciphylaxis [3]. Diabetic foot ulcers (DFU) are one of the most challenging complications due to high morbidity and associated mortality and precede the majority of non-traumatic lower limb amputations in the adult population [4–7]. Multiple factors, including neuropathy, ischemia, impaired immune function, and infection have been associated with DFU development and poor healing outcomes [4, 8] however, the molecular mechanisms that lead to inhibition of wound healing in diabetic population are still not well understood.

Patients with DM often develop macro and microvascular vascular problems [9], abnormal angiogenesis [10], humoral and cellular immune deficiencies, infection [11], extracellular matrix changes and fibrosis [12], nephropathy and neuropathy [9] among others. Most of these are commonly found in DFUs [13], but it is not clear if their role is causal or consequential in the impairment of healing.

Previously, we have shown the first evidence that micro-RNAs (miRs) contribute to inhibition of healing of human venous ulcers [14]. MiRs are small (~22 nucleotides) non-coding RNAs that can regulate gene expression post-transcriptionally, contributing to fine tuning of tissue homeostasis and function [15–18] as well as to various pathologic conditions [19, 20]. Recently, miRs were found de-regulated in human diabetic corneal tissue [21] suggesting their role in the impaired healing of this epithelium. Thus, DM may modify miR expression contributing to development of DFUs.

While many tissues are found to be profoundly affected by DM [9, 10, 12, 21], our understanding on how DM alters intact human foot skin has been limited. We postulate that DM may alter the skin phenotype and predispose it to poor healing. Therefore, we sought to investigate specific cellular and molecular differences in human diabetic foot skin that could potentially lead to the development of non-healing phenotype observed in DFUs. We obtained tissue specimens and primary cells from patients undergoing voluntary podiatric correction surgery and performed comparative genomics and immunohistochemistry analyses to identify differences between non-neuropathic diabetic (DFS) and non-diabetic (NFS) human foot skin. Surprisingly, we found only subtle differences in both miR and mRNA profiles. Specifically, there was a trend towards induction of miR 29c-3p in primary dermal fibroblasts generated from diabetic foot skin compared to non-diabetic foot fibroblasts, whereas miR-31-5p and miR-31-3p were induced in laser captured epidermis from diabetic patients. No discernable differences were observed in skin morphology and collagen deposition, as well as in proliferative capacity of epidermis, presence of lymphocytes and number of blood vessels in the dermis.

We conclude that human diabetic non-neuropathic foot skin shows minor differences at the transcriptional, miR levels, or tissue morphology compared to non-diabetic foot skin. These data suggest that, perhaps, additional DM-associated complications such as neuropathy, longer duration of DM, or vascular problems may have a more important role in development of DFU.

## Results

### miR Profiles of Epidermis and Dermal Fibroblast Show Small Differences between DFS and NFS

**Fig 1:**
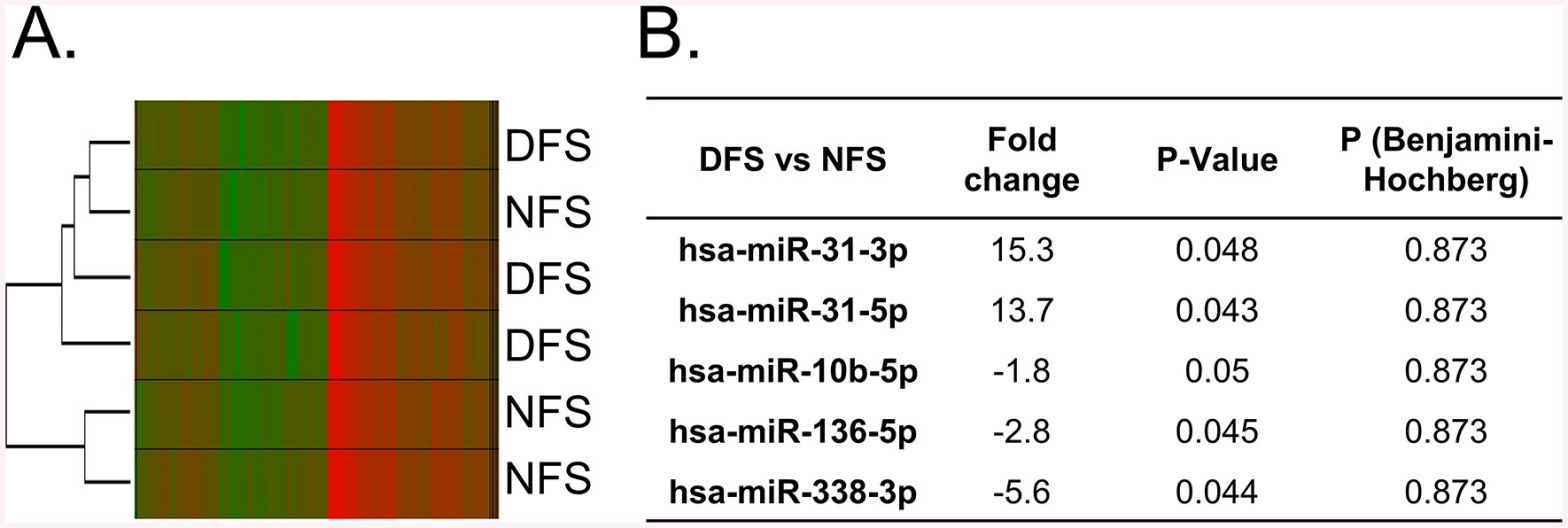
Differential expression of miRs reveals subtle changes between diabetic and non-diabetic foot epidermis. **A.** Clustering analysis of NFS and DFS, which groups samples based on how similar is their miR expression, revealed no specific segregation of NFS or DFS and consistenly the heatmap did not show any distinguishable pattern in miR expression between the groups. **B**. Five microRNAs were found significantly de-regulated in laser captured epidermis from DFS as compared to NFS (t-test, unadjusted p<0.05). However, none were found statistically significant after multiple testing correction (FDR<0.05).

To identify differentially expressed epidermal miRs between DFS and NFS that may compromise tissue healing capacity we used laser capture microdissection (LCM). The epidermal compartment containing keratinocytes was captured from 3 DFS and 3 NFS tissue specimens after morphology was evaluated as previously described [22]. Total RNA was isolated from the epidermis and miRs were profiled using Ready-to-Use PCR panels v3 which include 752 human miRs. After data processing and elimination of low expression miRs (Ct > 35) we detected 198 miRs expressed in the foot skin keratinocytes. Clustering and principal component analysis (PCA) did not show any specific distribution or segregation among the DFS and NFS groups (Fig 1A). Five miRs were found to be differentially expressed with statistical significance (unadjusted p-value <0.05). Two of these miRs corresponding to the same stem-loop precursor, miR-31-5p and miR-31-3p, were up-regulated by more than 10 fold while the other three (miR-338-3p, -136-5p and -10b-5p) were found to be down-regulated in DFS (Fig 1B). However, none of these miRs were significantly regulated when a multiple testing correction was applied (Fig 1B).

**Fig 2:**
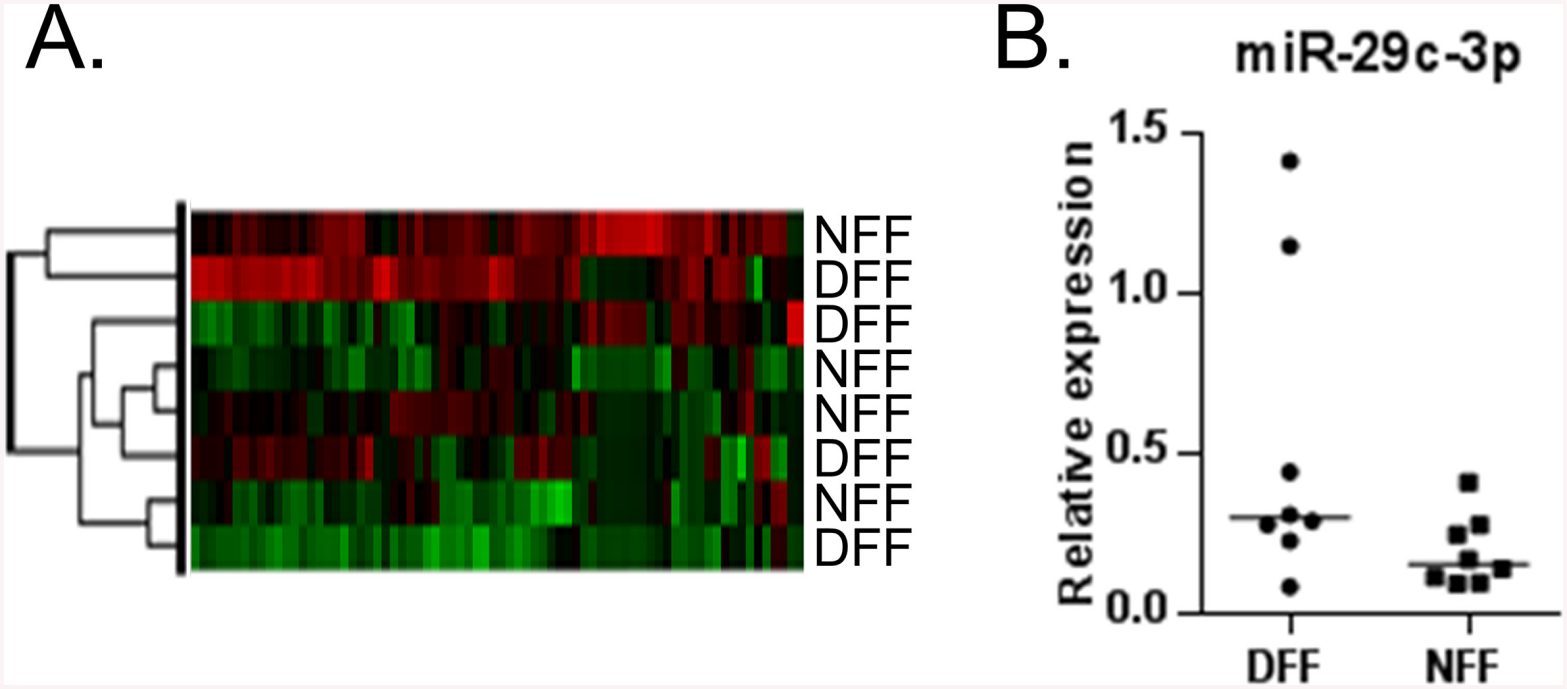
microRNA profiling of diabetic (DFF) and non-diabetic (NFF) foot fibroblasts shows induction of miR-29c. **A.** Primary cells were generated from samples of diabetic foot (n = 4) and non-diabetic foot skin (n = 4) and miR profiling clustering analysis of DFF and NFF (miRs de-regulated > 2 fold) shows no specific segregation of DFF and NFF, similar to the pattern seen in the epidermis. **B.** Relative miR-29c expression in a prospective set of samples shows a trend of induction in DFF compared to NFF (Median, n = 8 per group, t-test, p = 0.09).

Unlike epidermis which consists mostly of keratinocytes, fibroblasts are scarce in the dermis. This makes the LCM and RNA extraction in sufficient quantity and quality for miR profiling technically challenging. Therefore to assess differences between DFS and NFS in the dermal compartment, we generated primary fibroblasts from 4 DFS and 4 NFS tissue samples. MiR profiling was performed from the generated primary cells using nanoString nCounter miR Expression Assays. We found 22 miRs regulated greater than 2-fold between diabetic (DFF) and healthy non-diabetic foot fibroblasts (NFF). However, none of these miRs achieved statistical significance (data not shown). Similarly to miRs expressed in the epidermis, clustering analysis of dermal miRs did not reveal any specific segregation (Fig 2A). Nonetheless, we determined the expression of miR-29c-3p in a larger set of cell lines from 8 additional DFS and 8 NFS. MiR-29 family has been found to be up-regulated in different cells and tissues from diabetic patients as well as from diabetic rodent models [23]. Based on analyses from the larger sample set we found that miR-29c showed a trend of induction in DFF compared to NFF, however it was not statistically significant (p = 0.09). (Fig 2B).

### Validation of the Epidermal and Dermal miR Expression in the Skin

**Fig 3:**
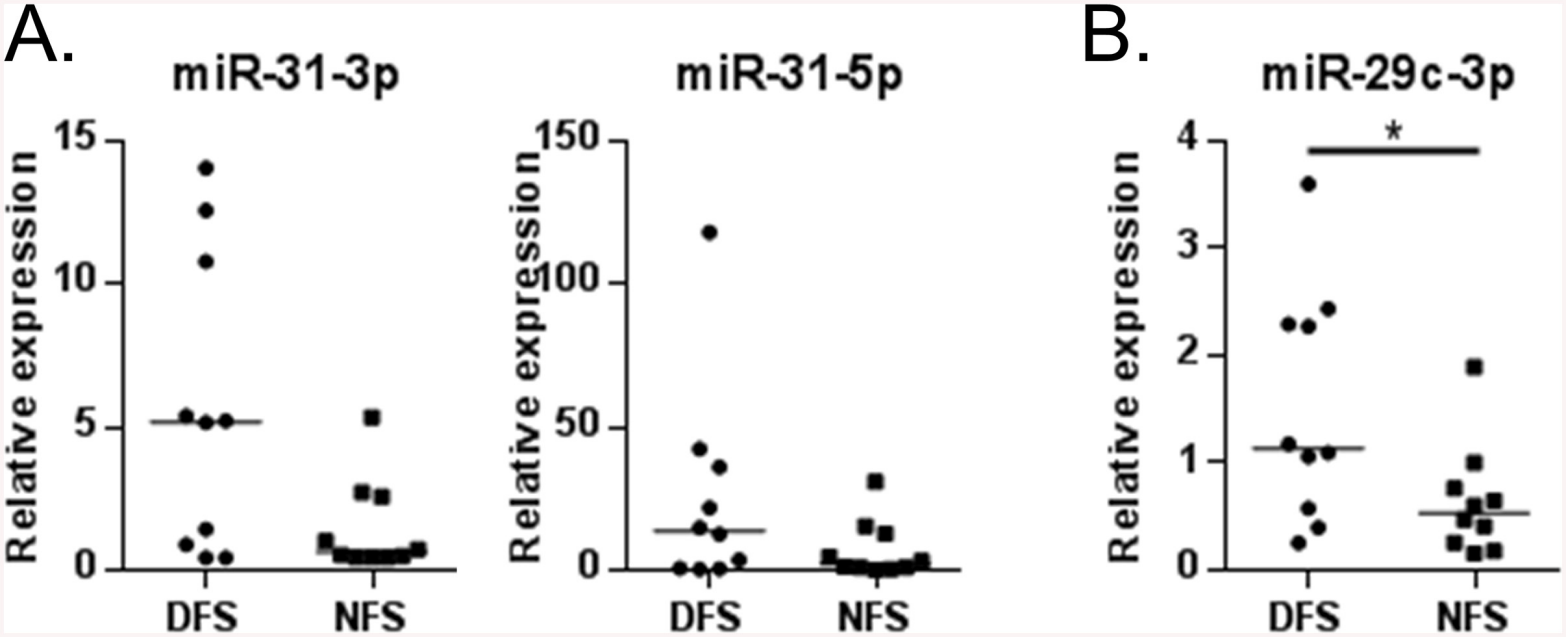
miR-31-3p, miR-31-5, and miR-29c-3p expression shows up-regulation in full thickness skin samples. **A.** Both miR-31-3p and miR-31-5p show up-regulation in DFS compared to NFS, similar to what was observed in the PCR arrays generated from epidermis. However, they did not reach statistical significance when tested in a larger set of full thickness biopsies (miR-31-3p p = 0.07, miR-31-5p p = 0.31). **B.** Relative miR-29c-3p expression in full thickness skin biopsies confirms statistically significant up-regulation in prospectively collected samples (p = 0.043). Graphs show median and sample distribution. N = 10 samples per group. Mann Whitney U test was performed to determine whether differences between groups were statistically significant. * p<0.05.

To validate the data from individual tissue compartments (epidermis and dermis) we examined the expression of the top induced miRs in the corresponding full thickness skin samples. We found a high correlation of miR-31-3p and miR-31-5p expression between the laser captured epidermis and full thickness biopsies (n = 6, miR-31-3p Spearman’s ρ = 0.94, p = 0.017; miR-31-5p Spearman’s ρ = 0.82, p = 0.072). We then tested the expression of these miRs in a larger set of prospectively collected skin samples, ten DFS and ten NFS, and found a high level of individual variability (Fig 3A). Even though there is a trend towards increased expression of both miR-31-3p and miR-31-5p in DFS in comparison to NFS, this difference did not reach statistical significance (p>0.05).

We also evaluated expression of miR-29c-3p in the same full thickness foot skin samples from ten diabetic and ten non-diabetic patients. miR-29c-3p was found to be significantly induced in diabetic skin, which correlated with its induction in diabetic foot fibroblasts (Fig 3B). Taken together, the miR analyses suggest that epidermal miRs show very subtle differences in expression between unwounded diabetic foot and non-diabetic foot skin, while diabetes correlates with induced miR-29c.

### Comparative Genomics between Non-Diabetic and Diabetic Foot Skin Reveals Minor Differences

**Fig 4:**
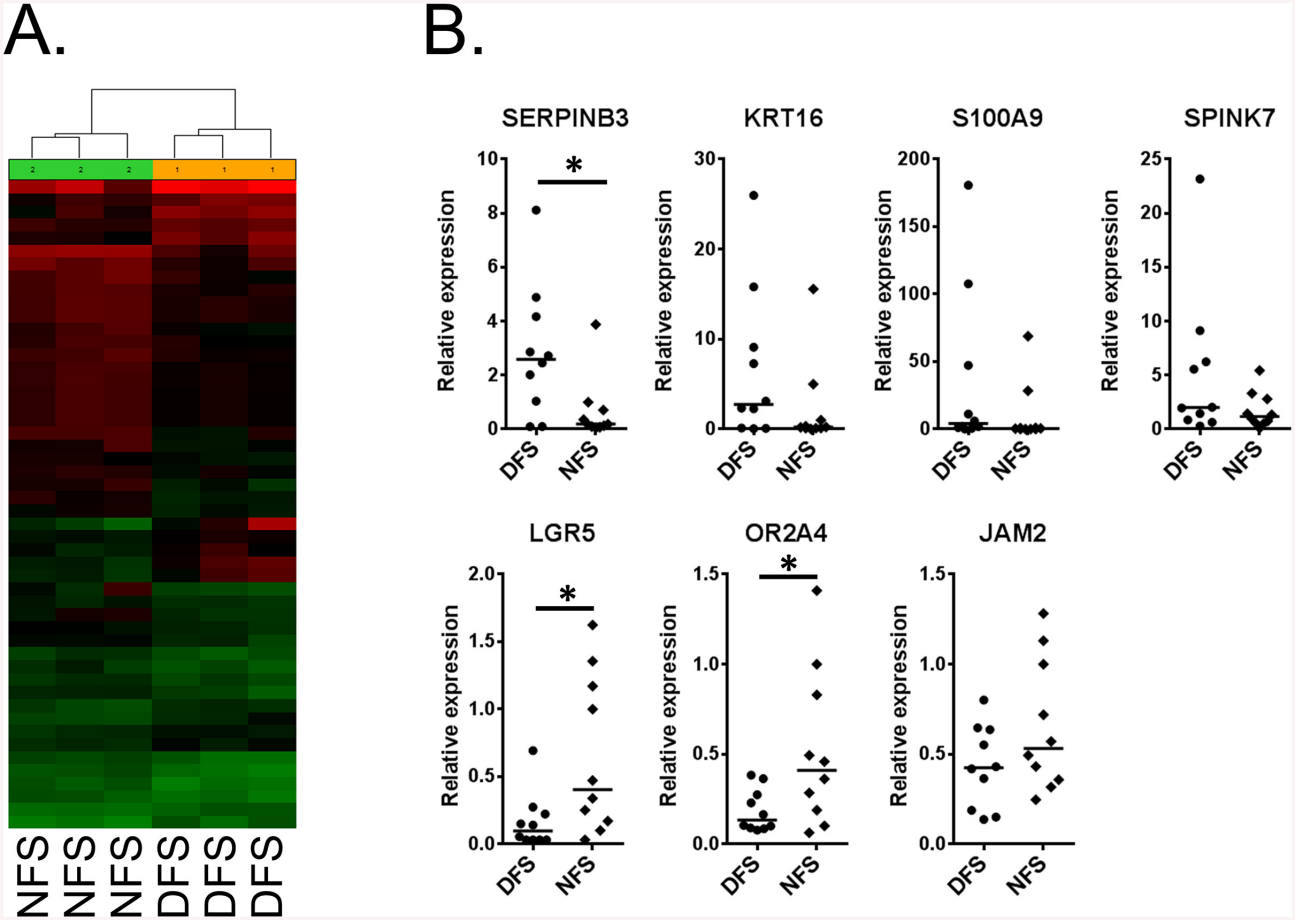
Differential gene expression between diabetic and non-diabetic foot skin reveals specific small sets of genes regulated in DFS. **A**. Heatmap showing the distinct clustering of NFS and DFS of differentially expressed genes. **B**. PCR validation of differentially regulated genes in full thickness biopsies. All the genes tested followed the same trend of up- or down-regulation observed from the microarray data, SERPINB3, LGR5, and OR2A4 reached statistical significance. Plots indicate sample distribution and median. (*p<0.05, N = 10 samples per group).

**Fig 5:**
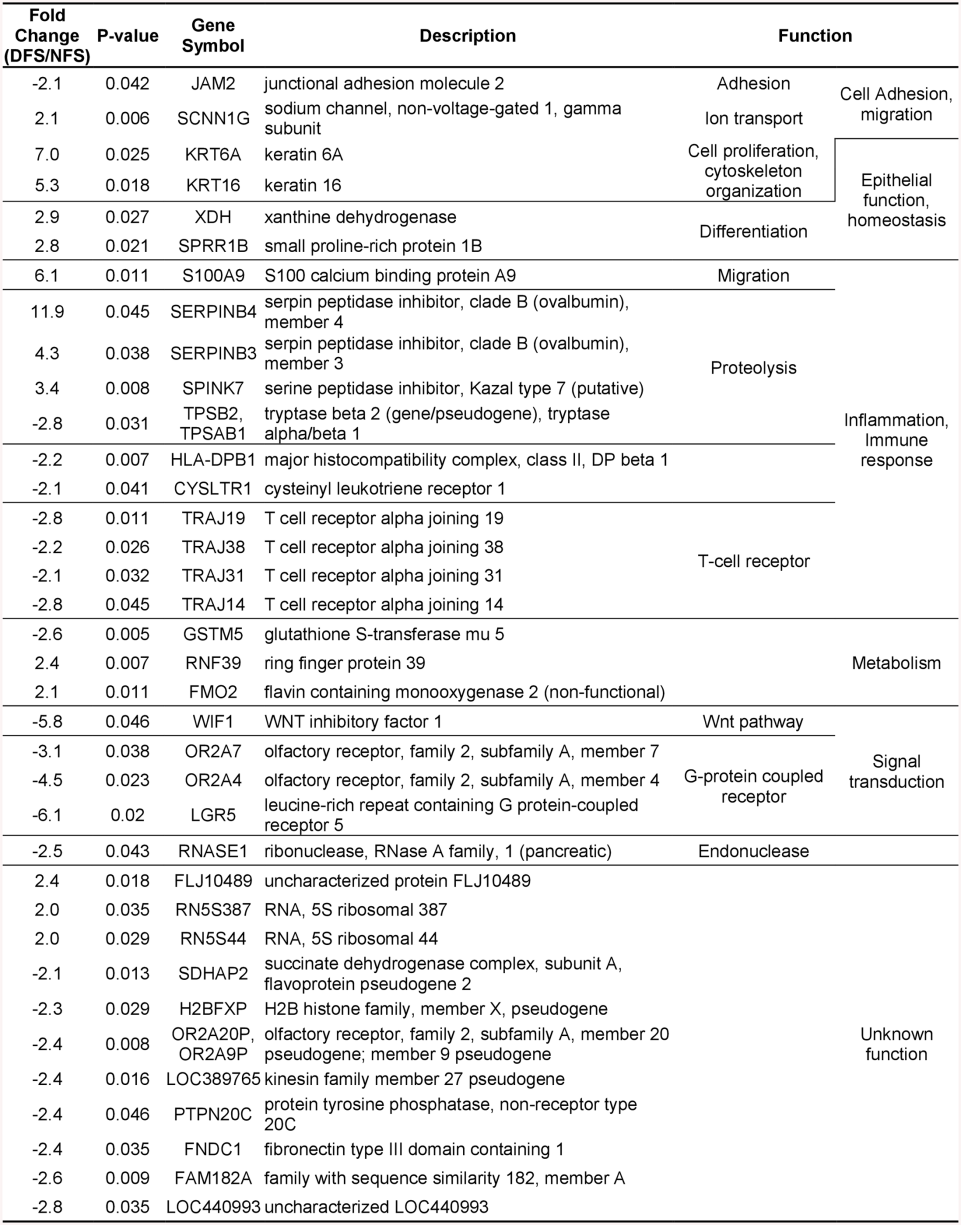
Differentially expressed genes between non-diabetic and diabetic full thickness skin biopsies. A list of genes that are regulated >2-fold in diabetic foot skin when compared to non-diabetic foot skin is presented along with their roles in different cellular processes.

Next we investigated differential gene expression between full thickness DFS and NFS using microarrays. We used the Affymetrix GeneChip Human Gene 2.0 ST Arrays. Of 40,716 transcripts, we detected 36 genes differentially expressed between DFS and NFS (>2 fold change, uncorrected p< 0.05). Unlike the miR profiles, the heat maps and cluster analysis revealed that DFS and NFS were grouped into distinct clusters (Fig 4A) suggesting the differential expression of these genes is sufficient to discern between the two groups. Fourteen genes were found up-regulated and twenty-two down-regulated in DFS compared to NFS, but none of these genes passed the multiple correction testing (Fig 5). Using a larger sample set we examined seven of the top regulated genes by qPCR and found Serpin Peptidase Inhibitor Clade B Member 3 (SERPINB3), Leucine-Rich Repeat Containing G Protein-Coupled Receptor 5 (LGR5), and Olfactory Receptor Family 2 Subfamily A Member 4 (OR2A4) to be differentially expressed in DFS versus NFS (n = 10 per group, p<0.05) (Fig 4B). S100 Calcium Binding Protein A9 (S100A9) did not reach statistical significance but showed a trend of induction in DFS (p = 0.06). These results indicate that there are subtle differences in the transcriptome of DFS compared to NFS.

### Histological Evaluation Shows No Observable Differences between DFS and NFS

**Fig 6:**
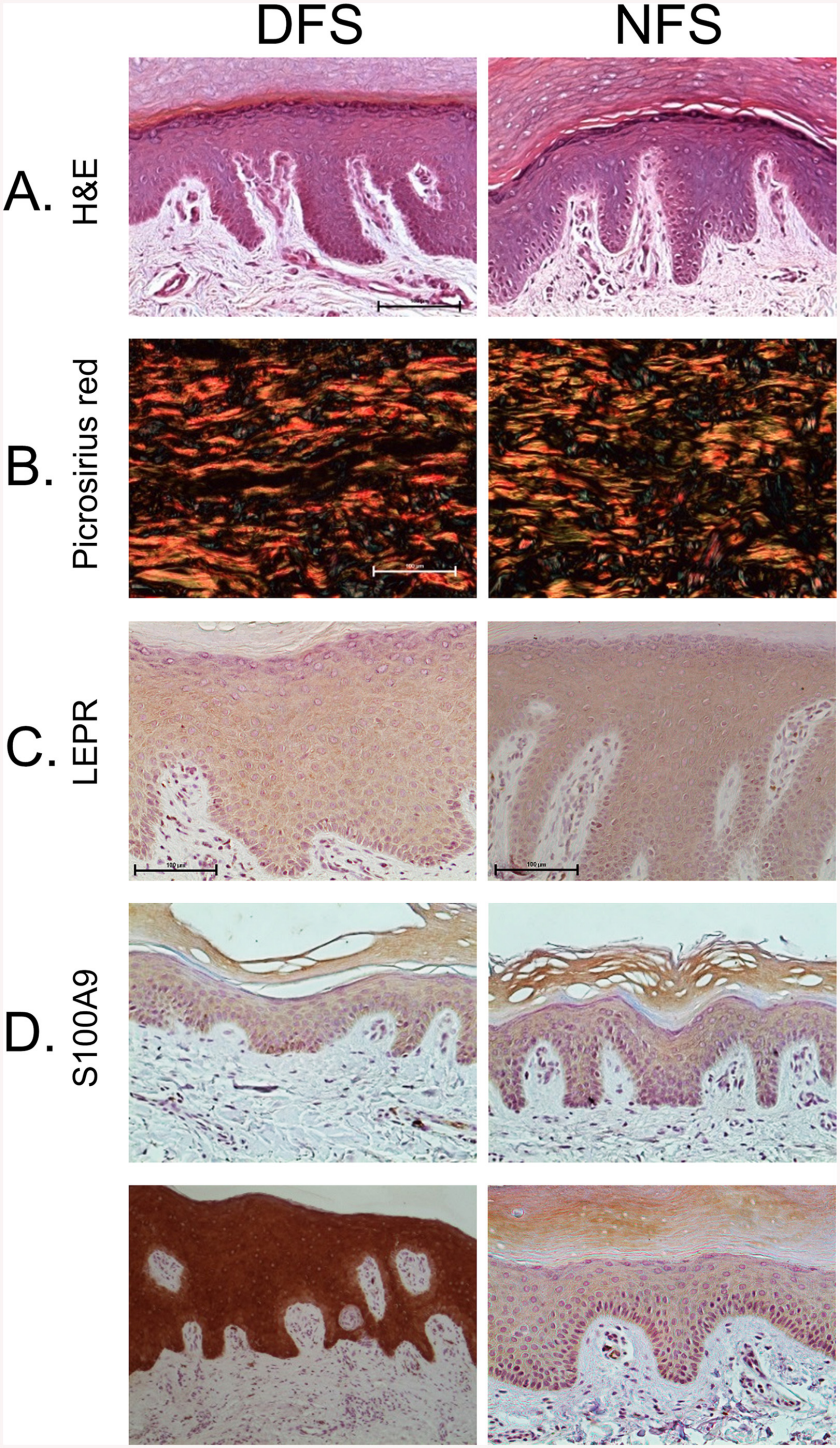
Morphological and immunohistochemical evaluation shows no substantial differences between DFS and NFS. **A**. Histological assessment by H&E staining shows similar skin morphology between DFS and NFS. **B**. Collagen fiber alignment and thickness assessed by picrosirius red staining under polarized light shows no difference between DFS and NFS. **C**. LEPR immuno-peroxidase staining shows signal throughout the epidermis of both DFS and NFS with no difference in localization or staining intensity. **D**. S100A9 immuno-peroxidase staining of DFS shows variable expression whereby 67% of DFS showed similar expression to NFS (upper panel), while the other 33% showed much greater expression compared to NFS (D, lower panel).

Normal plantar skin consists of a thick ridged epidermis with all typical epidermal layers identifiable along with a thick cornified layer. The histological evaluation of DFS did not show any observable difference in morphology or thickness when compared to NFS (Fig 6A). Given that changes in extracellular matrix (ECM), including collagen production [24], growth factors [25], and ECM remodeling proteins [26], have been reported in different tissues of diabetic animals [27, 28], we assessed collagen orientation and fiber composition by picrosirius red staining and polarized light microscopy. We did not observe any difference in fiber composition or orientation. The basket-weave orientation of collagen, characteristic of normal skin, was observed in both diabetic and non-diabetic foot skin (Fig 6B).

Based on its important role in wound healing we also investigated Leptin receptor (LEPR). A diabetic mouse model carrying a mutation in LEPR exhibits delayed healing and has been widely used as a model for impaired wound closure [28]. Furthermore, we have shown that LEPR is down-regulated in venous ulcers, another type of chronic wound [14]. However, immuno-peroxidase staining of LEPR showed staining throughout the epidermis of both DFS and NFS with a similar intensity (Fig 6C).

To verify the results obtained from gene arrays and qPCR showing up-regulation of S100A9 in DFS (Fig 4B), we performed immuno-peroxidase staining of S100A9. The majority of DFS tested (67%) showed similar S100A9 staining intensity as NFS (Fig 6D upper panel). However 33% of DFS exhibited more intense staining in the epidermis compared to NFS (Fig 6D lower panel). Based on these histological analyses, we conclude that DFS appear generally similar to NFS.

### Quantification of Lymphocyte, Blood Vessels, and Keratinocyte Proliferation Reveals No Difference between DFS and NFS

**Fig 7:**
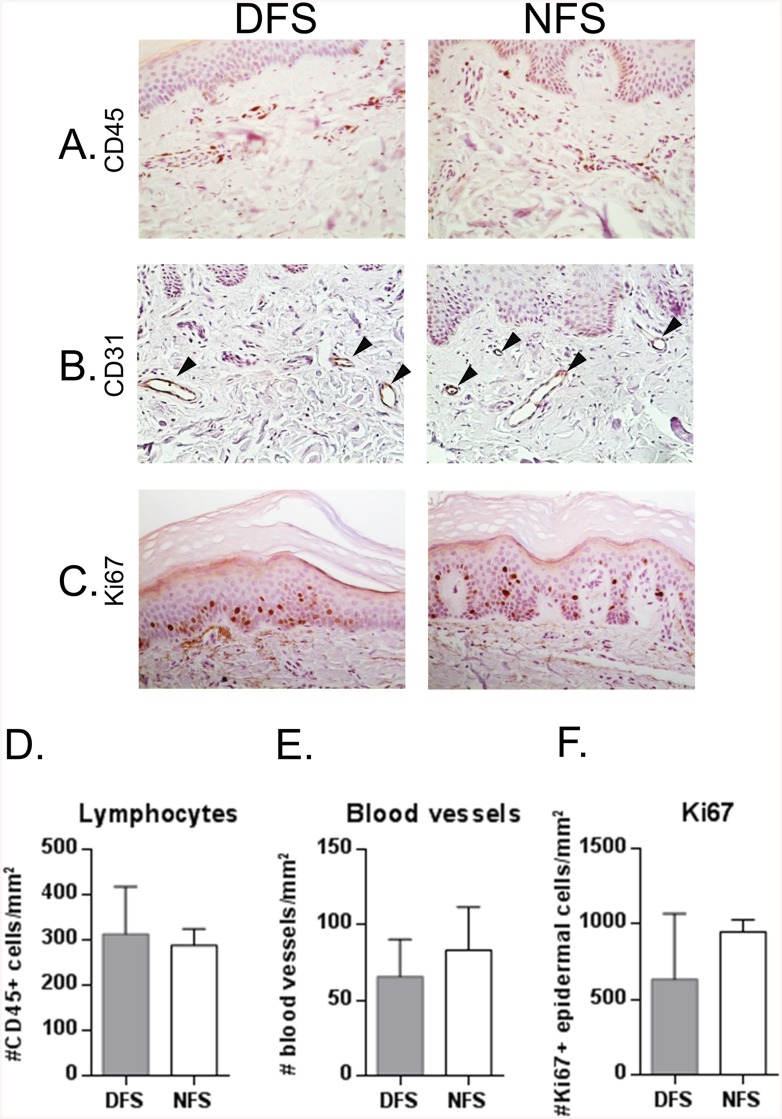
Similar numbers of lymphocytes, blood vessels and proliferating keratinocytes are found in DFS and NFS. Immunostaining and quantification for CD45, a lymphocyte marker, (**A, D**), CD31, an endothelial cell marker, (**B, E**) and Ki67, a proliferation marker (C,F), reveal no differences in the number of lymphocytes, blood vessels and proliferating keratinocytes found in DFS and NFS. Ki67 immuno-peroxidase staining for proliferating cells (**C**) shows that proliferative keratinocytes were located in the basal layer of the skin, as expected. No significant differences were found between DFS and NFS in numbers of CD45+ cells (**D**), number of CD31+ (**E**) or Ki67 positive keratinocytes (F). Bar graphs indicate mean and SD.

It is well known that diabetes is associated with immune dysfunctions as well as increased prevalence of infections [11]. For example, diabetes and uncontrolled hyperglycemia can reduce the number of circulating lymphocytes and increase their apoptosis [29]. Thus, we examined presence of lymphocytes in DFS and NFS by CD45 immunohistochemistry (Fig 7A and 7D). The quantification of CD45 positive cells revealed similar numbers of CD45^+^ lymphocytes in DFS and NFS.

Diabetic individuals also develop microvascular complications including retinopathy, nephropathy, and neuropathy due to abnormal angiogenesis or capillary function [9, 10]. Therefore, we quantified the number of blood vessels in the dermis using CD31 immuno-peroxidase staining (Fig 7B and 7E). Based on CD31 immunohistochemistry data and quantification we conclude that both diabetic and non-diabetic subject have similar numbers of blood vessels in the foot skin.

Hyperproliferation is one of the hallmarks of the non-healing chronic wound epidermis [30]. We evaluated keratinocyte proliferation in DFS and NFS tissue specimens by Ki67 immuno-peroxidase staining and determined the number of proliferating cells. DFS showed higher variability in the number of Ki67 positive cells however, no statistical differences were seen between DFS and NFS (Fig 7C and 7F). We conclude that DFS and NFS have comparable levels of lymphocytes, blood vessels, and proliferating keratinocytes.

In summary, careful examination of DFS reveals only subtle molecular, cellular, and tissue changes. Our findings suggest that there may not be an innate inability of diabetic skin to heal and that additional factors contribute to wound formation and may play a role in inhibition of healing observed in patients suffering from DFUs.

## Discussion

This work focused on examining features of non-neuropathic foot skin in individuals with DM compared to individuals without DM to identify if cutaneous differences could predispose diabetic patients to ulcer development and impaired healing. We evaluated differences due to diabetes in human full thickness skin biopsies, microdissected epidermis, and primary dermal fibroblasts, using a comprehensive approach that included transcriptional profiling, miR analyses, qPCR, and immunohistochemistry. Surprisingly, changes were subtle, suggesting that additional factors may contribute to the development of DFUs.

We encountered several technical challenges that resulted in the approach we describe. One would expect that remaining dermis after capture of epidermis by LCM could be used for assessment of dermal miR/mRNAs. However, LCM was performed from paraffin embedded sections from which primary cells could not be generated. We also attempted to grow primary keratinocytes, however, the growth potential is very low that did not yield sufficient biomaterial for evaluations. Therefore, we focused on LCM captured epidermis and primary fibroblast cultures generated from dermis as well as assessment of full thickness samples obtained from prospective patients for additional level of validation.

Assessment of differentially expressed miRs in both laser captured epidermis and primary fibroblasts derived from DFS and NFS samples revealed very few differences. Only 5 miRs were differentially expressed in the epidermis whereas none were found deregulated in fibroblasts. Out of the de-regulated epidermal miRs none were statistically significant after multiple testing correction (FDR>0.05). Further, when the top two up-regulated miR-31-5p and miR-31-3p were assayed in a larger sample set they did not reach statistical significance due to variable levels from patient to patient. Interestingly, most of the individuals with DM with the highest miR-31 levels also had renal insufficiency suggesting either longer lasting or more severe DM. In fibroblasts, miR-29c showed a trend of induction and was significantly up-regulated in full thickness biopsies, suggesting a possible role in regulating fibroblast functions in the diabetic environment. Lack of statistical significance of miR-29c may originate from several reasons. Most likely, it is the individual variability of its expression from cell lines within a group and relatively small sample size. Alternatively, although miR-29c was not found regulated in LCM of epidermis its regulation in fibroblast may still require cross talk with keratinocytes or other cell types found in the wound. Consistently, miR-29c has been reported to be induced in the liver, skeletal muscle, and fat of diabetic mice and to repress insulin-stimulated glucose uptake in adipocytes [23, 31]. Furthermore, miR-29b, another member of miR-29 family, has been shown to directly target Type I collagen in human dermal fibroblasts [32]. However, the implications of miR-29c up-regulation in diabetic skin require further investigation. Madhyastha et. al. had previously identified a microRNA signature in diabetic mice wound healing [33], however we did not observe any de-regulation of these miRs in human diabetic skin. A possible explanation for this, could be due to differences between species, or the multifactorial cause of diabetes in humans, which is not contemplated completely by the use of KKAY mice. Furthermore, none of the miRs discussed above were found in the diabetic mice because they were not covered by the arrays used in that work.

Patients with DM often exhibit delayed corneal healing following surgery or injury [21]. Recently, miRs were found differentially expressed in human corneas of DM patients [21], suggesting their role in impaired healing in this epithelium. This suggests that skin in general or foot skin in particular is less affected by DM, at least, at the miR level, than corneal epithelium. An alternative explanation is that the patients in this study, having not developed neuropathy, may represent a subset of patients with better controlled diabetes and/or shorter duration of diabetes compared to those who develop corneal disease.

It is noteworthy that individual variability within each group, DFS and NFS, was present in both skin samples and primary fibroblasts, underscoring the inherent differences in each patient’s skin. One can argue that small differences found in miR expression between DFS and NFS (in full thickness skin, epidermis, or fibroblasts) may have larger biological significance, since they show a trend of de-regulation, even though they did not achieve statistical significance. Given the power of regulation of miR-mediated gene expression that may simultaneously affect hundreds of transcripts, very small changes may have significant biological impact. Specifically, miR-31 was recently found to be up-regulated in psoriasis, which shares some phenotypic similarities with DFUs such as a hyperproliferative epidermis, parakeratosis, and unresolved inflammation. In psoriasis, miR-31 targets serine/threonine kinase 40 (STK40), a negative regulator of NFkB, which in turn results in increased expression of proinflammatory cytokines and chemokines [34]. We have shown that miR-31 sustains an activated keratinocyte phenotype [35] and has been found to regulate cell differentiation and proliferation, all of which are de-regulated in DFUs [36, 37]. Yet, despite showing a trend of induction of miR-31 in DFS we did not observe either morphological changes in terms of differentiation or inflammation in the epidermis, or increased expression of the proliferation marker Ki67. Furthermore, miR-31 was not predicted to target any of the down-regulated genes, but the possibility of it affecting target genes post-transcriptionally without reducing the mRNA levels cannot be excluded.

Global transcriptome analysis of full thickness biopsies comparing DFS and NFS showed findings similar to the miR profiling. Only 36 genes were found differentially expressed in DFS compared to NFS, and none passed the multiple testing correction. Among these 36 genes there are several clusters of genes with specific functional categories. For example, among the suppressed genes we found multiple genes encoding T cell receptors, suggesting the possibility of abnormal T cell function in diabetic skin. In addition, the observed trend in suppression of β tryptases (TBSP2) may suggest a lack of mast cells, as β tryptases appear to be the main isoenzymes in mast cells [38]. Validation of seven de-regulated genes by qPCR demonstrated statistically significant differences in expression of SERPINB3, LGR5, and OR2A4 whereas S100A9 was nearly significant (p = 0.06). Both SERPINB3 and S100A9 are modulators of inflammatory response [39–41], highly inducible in keratinocytes, and have been found to be de-regulated in inflammatory skin diseases such as venous ulcers [42], psoriasis [41, 43], atopic dermatitis [39, 41], and skin cancer [40]. SERPINB3/B4 have been suggested to play a role in initiation of the acute inflammatory response, partly due to regulation of S100A8/A9 [41]. S100A8/A9 are damage-associated molecular pattern (DAMP) molecules normally expressed at low levels in keratinocytes but can be induced by variety of physical stresses or cytokines. They dimerize, and upon induction can up-regulates pro-inflammatory cytokine and chemokine expression and also keratinocyte proliferation possibly functioning as a positive feedback mechanism [44]. Staining for S100A9 in full thickness skin biopsy sections revealed high individual variability within the DFS samples, with some patient showing similar expression to NFS, while other DFS showed high expression throughout the epidermis indicating that diabetic skin may be prone to inflammation.

The olfactory receptor, OR2A4, was down-regulated in DFS compared to NFS. OR2A4 is a G protein coupled receptor, whose functions are mostly uncharacterized. While Olfactory receptors have been shown to play a role in cytokinesis [45] and chemosensing in tissues such as renal epithelium [46], their involvement in wound healing and diabetes is not known. Another G-protein coupled receptor LGR5, known for its ability to potentiate the canonical Wnt signaling pathway [47], was also found suppressed in DFS compared to NFS. LGR5 is known as a hair follicle stem cell marker [48], however its role in volar skin has not been described yet.

It remains possible, that observed subtle differences in mRNA/miRs between DFS and NFS may have biological significance and may point to a subgroup of patients with DM with higher glucose levels or longer history of diabetes that are more likely to develop neuropathy, arterial disease, ulcers or slow healing ulcers, however, a much larger population studies are needed to better characterize such subset of patients.

The skin architecture was also comparable between DFS and NFS. The epidermis of DFS showed no evidence of any of the morphological characteristics of DFUs, such as hyperproliferative epidermis, parakeratosis, hyper-keratosis, or fibrosis. The dermis lacked observable differences in its morphology, collagen orientation, and other markers. Together these observations suggest that unwounded skin from diabetic patients without neuropathy is not highly predisposed to develop DFUs.

Although subtle changes in DFS may not predispose for DFU development, these changes may contribute to the inability of DFS to mount an appropriate response to wounding. For instance, we found no difference in LEPR expression in the epidermis of unwounded DFS, although LEPR mutant mice (*db/db*) have been widely used as a model of diabetes and impaired wound healing. In addition, we have previously shown that LEPR is down-regulated in venous ulcers of non-diabetic patients [14]. This suggests that the loss of LEPR is not necessary for the development of a chronic wound, but rather its down-regulation impedes healing when a wound has already occurred. Thus, the down-regulation of LEPR in human ulcers could be a consequence rather than a cause of ulceration.

The quantification of the number of blood vessels and lymphocytes in the dermis of DFS did not show any differences either. Consistently with this, Haemmerle et al, did not find differences in numbers of blood vessels and lymphocytes between diabetic and non-diabetic skin [49]. They reported increased lymph vessel density in diabetic skin and gene expression changes in lymphatic endothelial cells with functions related to inflammation, lymphatic vessel remodeling and lymphangiogenesis among others [49]. A meta-analysis of that study identified several miRs potentially involved in those changes [50], none of which were found de-regulated in this study suggesting they could be cell-type specific. Interestingly, a published study shows an increased number of blood vessels and lymphocytes in forearm skin from neuropathic diabetic individuals [51]. It is possible that these changes are consequence of diabetic-related neuropathy rather than diabetes itself. However, DM or the presence of neuropathy did not significantly affect healing in a randomized controlled clinical trial of 228 patients with venous ulcers [52], further supporting the notion that DM may not predispose for poor cutaneous healing.

Taken together, these data demonstrating only subtle differences between DFS and NFS suggest that DFS may not be predisposed to ulcer development and further points to other factors such as glucose control, duration of DM, neuropathy or additional diabetic complications as important factors in the development and poor healing associated with DFUs. The fact that there are only subtle differences between DFS and NFS implies that preventive care including frequent assessments of neuropathy and vascular supply could reduce the incidence of DFUs, improve the quality of life of the diabetic population, and reduce overall healthcare costs.

## Materials and Methods

### Tissue Samples

Both non-diabetic and non-neuropathic diabetic (type 2) skin specimens for epidermal profiling (LCM, mRNA/miRNA) were obtained as discarded tissue (specimens were obtained from debridement procedures and otherwise would be discarded) from patients undergoing podiatric surgery at the University of Miami Hospital and, as such, was found to be exempt under 45 CFR46.101.2 by the IRB at the University Of Miami Miller School Of Medicine. Specimens did not contain any of the 18 identifiers noted in the privacy rule and therefore no informed consent was obtained. The specimens from which primary fibroblast cultures were generated were collected under the IRB approved protocols IRB# 20120473 and IRB# 20120574 specifically for the project and informed consent was obtained from the subjects prior to specimen collection. Our laboratories generated primary fibroblast cultures following previously published protocol [53] also, please see the section “Isolation of fibroblasts derived from patients” below for more details)). Demographics of patients are presented in Table 1. Samples were considered as non-human subject research by the University of Miami Intuitional Review Board and thus did not require a signed consent. Verbal consent was approved by IRB and was not documented. Skin biopsies were either stored in RNA later (Applied Biosystems, Carlsbad, CA, USA) for subsequent RNA isolation or fixed in formalin for paraffin embedded.

**Table 1:** Patient demographics and sample information.

TYPE	N	Age±SD	Gender (M/F)	Etnicity (AA/H/HW/W)
**DFS**	10	62.2±7.6	6/4	2/1/5/2
**NFS**	10	53.6±15.1	5/5	2/0/7/1

AA = African-American, H = Hispanic, HW = Hispanic White, W = White

### Laser Capture Microdissection (LCM) and MicroRNA Profiling of Epidermis

Between 16 and 20 8–10μm sections were cut from the formalin-fixed paraffin embedded tissue blocks of non-diabetic and diabetic foot skin, placed on Arcturus PEN-membrane glass slides (Life Technologies, Carlsbad, CA, USA) and dried at 37°C for 1–2 hours. LCM was carried out on a Arcturus Veritas laser capture microdissection instrument and the epidermis was collected on CapSure Macro LCM Caps (Life Technologies). The caps were transferred to a tube containing 60μl of deparaffinization buffer (QIAGEN Inc., Valencia, CA, USA) and total RNA, including the microRNA fraction, was extracted using the FFPE miRNeasy kit (QIAGEN Inc.) according to the manufacturer’s instructions.

Total RNA concentration of the samples was quantified using NanoDrop 2000 (NanoDrop products, Wilmington, DE) and the RNA quality of these samples was determined by RT-qPCR of SNORD48 and miR-21 using the commercially available platforms miRCURY LNA (Exiqon, Woburn, MA, USA) or Quanta qScript microRNA Quantification System (Quanta BioSciences, Inc., Gaithersburg, MD, USA). The miR profiles for the epidermis of 3 NFS and 3 DFS, were generated using the miR Ready-to-Use PCR panels V3 (Exiqon) following the manufacturer’s specifications. The Ct values were normalized to the stably expressed reference gene SNORD49 using the Exiqon GenEX software and the expression levels in the NFS and DFS were compared. The qPCR profiling raw and normalized data are publically available in GEO database under the super series GSE68186.

### RNA Extraction and Quality Control

Total RNA including the miR fraction was extracted from the samples using the QIAGEN miRNeasy mini kit and following the manufacturer’s instructions. The RNA quality was assessed using the AGILENT bioanalyzer (Agilent Technologies, Palo Alto, CA, USA) to estimate the RNA integrity number (RIN). Samples with a RIN higher than 5 were used for mRNA profiling as described below.

### mRNA Profiling

We previously described methods for tissue specimen preparation and hybridization [42, 54, 55]. All processing and analysis of microarrays utilized standard protocols at the University of Miami Microarray Core Facility. Briefly, between 100 to 300 ng of total RNA was reverse transcribed, amplified, then the sense strand cDNA synthesized, labeled, and hybridized on arrays. The amplified, fragmented and biotin-labeled cDNAs were hybridized to the Affymetrix GeneChip Human Gene 2.0 ST microarray according to the manufacturer’s recommendations. Arrays were washed and stained using Affymetrix Fluidic stations 450 and scanned using Affymetrix GeneChip scanner 3000 7G. Image analysis was performed using the Affymetrix Command Console Software (AGCC). Resulting CEL files was imported into Expression Console Software (Affymetrix, Santa Clara, CA, USA) and underwent gene level normalization and signal summarization. The output files from this step were imported in Transcriptome Analysis Console (TAC) 2.0 Software (Affymetrix, Santa Clara, CA, USA) to identify differentially expressed genes and carry out clustering analysis. Only genes with a p-value lower than 0.05 and a fold-change greater than 2 were confirmed by qPCR in a prospective set of 10 NFS and 10 DFS. Microarray profiles are publically available in GEO database under the super series GSE68186.

### Isolation of Fibroblasts Derived from Patients and miR Profiling

Tissues were thoroughly washed in DMEM supplemented with 2× Penicillin/Streptomycin /Fungizone, and gentamicin (50mg/L). After, the epidermis was removed from specimens, the dermis was finely minced and placed in 12-well plates. For the establishment of fibroblast cultures, DMEM (Life Technologies) supplemented with 10% FBS, HEPES (1.9mg/ml), streptomycin (100 μg/ml), penicillin (100 U/ml), and Fungizone Antimycotic (0.25 μg/mL) was used [53]. RNA was isolated from cells (passage 1 or 2) using miRNeasy Mini Kit (QIAGEN).miR expression profiles of DFF and NFF fibroblasts were generated by nanoString nCounter miR Expression Assays (NanoString Technologies, Seattle, WA, USA) and analyzed using nSolver2.0 software. Data were normalized to gene controls included in the assays. Nanostring profiles are publically available in GEO database under the super series GSE68186.

### Real-Time qPCR MicroRNA and Gene Expression Analysis

The qScript microRNA Quantification System (Quanta BioSciences, Inc., Gaithersburg, MD, USA) was used for microRNA expression. Briefly, cDNA was synthesized from total RNA including the microRNA fraction using the qScript microRNA cDNA Synthesis Kit. 50pg of initial RNA were used per PCR reaction on a CFX Connect Real-Time PCR Detection System (Bio-rad, Hercules, CA, USA). SNORD48 was used as a reference gene to normalize the miR expression. For gene expression, cDNA was made with qScript cDNA Synthesis kit (Quanta BioSciences Inc., Gaithersburg, MD, USA). ARPC2 was used as a reference gene for normalization.

All real-time PCR reactions were done in triplicate using the PerfeCTa SYBR Green SuperMix (Quanta BioSciences) and the relative gene expression was calculated with the ddCT method.

### H&E, Immuno-Peroxidase, and Picrosirius Red Staining

The formalin fixed, paraffin embedded tissue was cut in 7 μm sections using a microtome. Slides containing sections were deparaffinized with xylene (EMD, Gibbstown, NJ, USA), rehydrated, and hemotoxylin and eosin stained or further processed for immuno-staining as described previously [42]. Endogenous peroxidase activity was quenched with 0.3% H_2_O_2_ in methanol and washed with distilled water. The slides were then incubated in Dako target retrieval solution (Dako, Carpinteria, CA, USA) for 30 minutes at 95°C for antigen retrieval, allowed to cool down, and then treated with Background punisher (MACH1 kit, Biocare Medical, Concord, CA, USA). Primary antibodies were diluted in 5% bovine serum albumin (Sigma-Aldrich, St. Louis, MO, USA) in TBS and applied to the samples (LEPR, Abcam cat # ab5593, 1;1000, CD31, Adb Serotec cat # MCA1746GA, 1:25; CD45, Dako cat # M0701, 1:100; Ki67, Abcam cat # ab15580, 1:500; S100A9 Abcam cat # 22506, 1:100). All antibodies were incubated overnight at 4°C, except for the anti CD45, which was incubated for 30 min at room temperature. The detection and chromogenic reaction was carried out using the MACH 1 Universal HRP-Polymer Detection system (Biocare Medical, Concord, CA, USA) and following manufacturer’s instructions. Picrosirius red (Electron Microscopy Sciences, Hatfield PA, USA) staining was carried out following manufacturer’s instructions. All slides were analyzed with a Nikon Eclipse E 400 microscope and digital images were obtained using a Qimaging camera and NIS-Elements BR3.10 software.

Quantification of CD45 positive cells, Ki67 positive cells, and CD31 positive blood vessels was performed using ImageJ software (NIH, Bethesda, NJ, USA). Briefly five (20X) images of each section were taken randomly and the positive cells or structures were counted within a rectangular dermal area of 0.046 mm^2^ (1000x500 pixels). For Ki67 quantification, positive cells were counted from the total epidermal section and normalized to the epidermal area.

### Statistical Analyses

Data were analyzed using the software Prism (Graphpad, La Jolla, CA, USA). Statistical significance between groups was determined using Mann-Whiteny U-test or Student’s t-test. A difference between groups was considered significant when p-value ≤ 0.05.
